# TRANSCRANIAL ELECTRICAL STIMULATION AS A TOOL TO UNDERSTAND AND ENHANCE MOBILITY IN OLDER ADULTS

**DOI:** 10.1093/geroni/igad104.0935

**Published:** 2023-12-21

**Authors:** Brad Manor

**Affiliations:** Hebrew SeniorLife/Harvard Medical School, Amherst, Massachusetts, United States

## Abstract

Standing and walking are complex cognitive-motor tasks that rely on the function of numerous brain networks. This reliance upon supraspinal elements of the motor control system increases with age and many age-related diseases, especially when an individual must navigate unfamiliar environments and/or simultaneously perform cognitive tasks like talking, reading signs, or making decisions. Noninvasive transcranial electrical stimulation (tES) can safely and selectively induce both acute and longer-term changes in brain network function. It thus enables cause-and-effect study of the motor control system and moreover, holds promise as a therapeutic strategy to counteract age- and disease-related alterations in standing, walking, and mobility. The purpose of this talk is to introduce the fundamentals of tES, describe the effects of tES on the neural control of mobility in older adults, and finally, discuss current limitations and related avenues for future research and development within this rapidly-growing field of study.

